# Thirty-five years of family medicine training and development in Uganda

**DOI:** 10.4102/phcfm.v17i1.4915

**Published:** 2025-05-22

**Authors:** Innocent K. Besigye, Brenda Tusubira, Michael Mulowooza, Fred Ndoboli

**Affiliations:** 1 Department of Family Medicine, School of Medicine, College of Health Sciences, Makerere University, Kampala, Uganda; 2 Department of Internal Medicine, St. Francis Hospital Nsambya, Kampala, Uganda; 3 Department of Community Health, Jinja Regional Referral Hospital, Jinja, Uganda; 4 Department of Public Health, School of Graduate Studies, Bugema University, Luwero, Uganda

**Keywords:** family medicine, family physicians, primary care, medical education, training

## Abstract

In Uganda, family medicine training was started in 1989 to train an all-round generalist able to provide comprehensive care at a district hospital. Since then, the training programme has undergone several changes to cater for the needs of communities in a changing world. Very low numbers of applicants and poor understanding of the discipline have been a persistent challenge. Availability of non-family physician champions, family physician role models and collaborative networks are key to development of family medicine.

## Introduction

Uganda is a landlocked country in East Africa with an estimated population of 46 million people.^1^ Uganda’s health policy is based on primary health care (PHC).^2^ There is a two-tier health system with PHC under the district local government and specialised services under the Ministry of Health (MOH).^3^ However, the MOH is responsible for financing, policy and planning of the overall health system and provision of leadership.^2^ Each hospital has a community health department responsible for providing primary care to the surrounding communities and strengthening the linkages between the hospital and the community. Health services are also offered by private providers (including the not-for-profit faith-based organisations and individuals for profit facilities) through public–private partnerships. The government of Uganda has a goal of every citizen living within a 5 km radius of a health facility.^4^

In Uganda, primary care as the service delivery component of the PHC approach is provided at five levels: Health Centre I–HCI (the village health team, consisting of a team of volunteer community members), Health Centre II–HCII (the community dispensary serving a ward or parish), Health Centre III–HCIII (serving a sub-county), Health Centre IV–HCIV (serving a county) and the district (community) hospital acting as a primary care referral centre.^4^ Currently, there are 216 HCIV facilities and 50 community hospitals in the country providing general medical and surgical services. Infectious diseases form the majority disease burden amid the rising prevalence of non-communicable diseases.^5^ A total of 12 medical schools provide training for Bachelor of Medicine and Bachelor of Surgery (MBChB) programme graduating medical officers (doctors without postgraduate training in family medicine) to work in primary care. Paramedical and nursing schools training other cadres of the PHC team also exist all over the country.

The different levels of health care provide different packages of primary care services. However, the primary care services should be accessible, comprehensive and coordinated provided with continuity and person-centredness. In Uganda, primary care quality is sub-optimal and the services lack comprehensiveness, coordination and continuity, but is strongly person-centred.^6^

### Background

Specialised family medicine training started in 1989 with a Master of Medicine in Community Practice.^7^ The goal was to train an all-round generalist able to provide comprehensive services, including surgical care at district hospitals. Three key stakeholders were involved in the preparatory phase of the programme: Makerere University, the Ministry of Health and the Memorial University of Newfoundland in Canada. The Canadian Development Agency provided funding for the initiative.

In 2005, the programme’s name was changed from Community Practice to Family Medicine to align with similar programmes in other parts of the world. This name change was greatly informed by Ugandan family physicians’ interactions with their international colleagues. Family physicians in Uganda through their national professional association have been part of global and regional bodies, associations and networks including the World Organization of Family Doctors (WONCA) and the Primary Care and Family Medicine (Primafamed) network, among others. This greatly assisted the development of the discipline through collaborative learning and sharing experiences.

Family physicians are recognised as specialists with clearly designated employment positions in the public sector and are paid at the same level as other specialists.^8^ Family physicians have clearly defined career path in the public sector but it is still unclear in the private sector. The only drawback is the misnomer of being employed as public health specialists, an issue that is being handled by the relevant stakeholders. Most family physicians trained in Uganda stayed and are employed in both public and private settings within the health services, academic institutions and non-governmental organisations. In all these settings, they provide clinical care, leadership and clinical governance as well as training of other health professionals.^9^ They work collaboratively with other medical specialists to address the comprehensive health needs of individuals, families and communities. However, their country coverage is still limited as there are currently only 70 family physicians for the whole Ugandan population across 146 districts. The goal is to have at least 2 family physicians in each district within the next 10 years.

## Training

Family medicine training started as a postgraduate programme. Currently, there are two family medicine training programmes in Uganda: at Makerere University and Mbarara University of Science and Technology. These institutions graduate an average of five family physicians per year. A third programme was started in the private sector at the Clarke International University (CIU) in the early 2010s as a part-time programme targeting practising doctors with no postgraduate qualification as potential trainees. Its approach was mainly work-place based learning and assessment using the trainee’s workplace. Unfortunately, the programme closed after graduating two cohorts of students.

The curricula for the training programmes have undergone multiple revisions to cater for new developments and updates in family medicine and primary care. These regular reviews ensure that the graduates are fit for purpose to address health system challenges in a changing world. The revisions have re-oriented the curriculum towards community-oriented primary care with the graduates able to integrate clinical care with public health in order to meet the diverse health needs of the populations and communities across the life course. In the early years of the programme, most of the teaching was conducted by other specialists because of the scarcity of family physicians. This has slowly changed with an increase in the number of family physicians choosing their careers in academic medicine. Most of the training is now conducted by family physicians within the district, which helps the trainees to acquire the principles and values of family medicine. The final assessment for knowledge and skills competence to provide primary care is done by individual universities. After university graduation, specialist licensure is done by the Uganda Medical and Dental Practitioners’ Council, the regulatory body of medical and dental practice, on presentation of academic documents with no further assessment.

**FIGURE 1:**
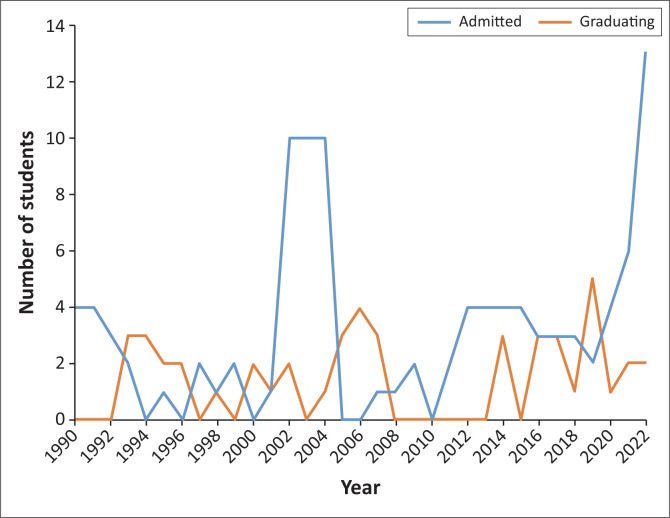
Number of family medicine applicants and graduates at Makerere University from 1990 to 2022.

Recruitment of trainees has been the main challenge. Very few doctors choose careers in family medicine and primary care. This is probably because of poor awareness and understanding of the discipline as well as the health system’s focus on hospital and specialist care. Figure 1 shows the number of applicants and graduates at Makerere University from 1990 to 2022. The average annual intake has been five trainees and a graduation rate of about four per year. There is a recent sudden increase in the number of family medicine applicants. This might be a response to the recently created family physician posts at all levels of hospitals in the outpatient and community health departments of public sector hospitals. A similar increase in the number of applicants occurred between the year 2000 and 2005 when the Uganda MOH, in an attempt to increase the number of family physicians, committed to fund all admitted trainees for that particular period.

Several medical schools in Uganda have introduced family medicine in the undergraduate curriculum. This was done to address the lack of primary care competences among graduates.^10^ However, the time dedicated in the curriculum for family medicine is limited given the breath of the content to be covered. Implementation of the course is also limited by the heavy biomedical orientation of the curriculum with its delivery in a tertiary care teaching hospital. In addition, no internship placement happens in primary care; yet when doctors complete a 12-months internship in tertiary care settings, they are expected to work in primary care.

## Lessons learnt

Advocacy for family medicine is a continuous activity mainly spearheaded by committed champions who may be family physicians or non-family physicians. Alliances with supportive non-family physician specialists are helpful.

Continuous review and revision of curricula to match the current updates and advances in family medicine and primary care is necessary. This will ensure that the training addresses the needs of society in a changing world.

Family physicians can motivate doctors to choose family medicine as a career path by being excellent role models in clinical practice. Most applicants for the family medicine training come from districts and/or facilities where family physicians are working.

The existing training programmes can help initiate programmes at other medical schools by sharing curricula, teaching materials and other relevant activities such as faculty development. The department of family medicine at Makerere University has supported three medical schools to start and implement undergraduate family medicine. The department is also helping the CIU to restart postgraduate family medicine training which had started and closed.

Networking with international organisations and regional networks provides a platform for collaboration and co-learning in the process of developing family medicine programmes. Such organisations and networks provide opportunities for educational and research capacity development relevant for faculty development. They also provide collaborative opportunities in research, student exchange and external examiners.
